# No evidence of early head circumference enlargements in children later diagnosed with autism in Israel

**DOI:** 10.1186/s13229-017-0129-9

**Published:** 2017-03-23

**Authors:** Ilan Dinstein, Shlomi Haar, Shir Atsmon, Hen Schtaerman

**Affiliations:** 1 Psychology Department, Ben Gurion University, Beer Sheva, 84105 Israel; 2 Cognitive and Brain Sciences Department, Ben Gurion University, Beer Sheva, 84105 Israel; 3 Child Development Center, Maccabi Health Services, Beer Sheva, 84893 Israel

**Keywords:** Autism, Head circumference, Biomarker, Early detection, Neuroanatomy

## Abstract

**Background:**

Large controversy exists regarding the potential existence and clinical significance of larger brain volumes in toddlers who later develop autism. Assessing this relationship is important for determining the clinical utility of early head circumference (HC) measures and for assessing the validity of the early overgrowth hypothesis of autism, which suggests that early accelerated brain development may be a hallmark of the disorder.

**Methods:**

We performed a retrospective comparison of HC, height, and weight measurements between 66 toddlers who were later diagnosed with autism and 66 matched controls. These toddlers represent an unbiased regional sample from a single health service provider in the southern district of Israel. On average, participating toddlers had >8 measurements between birth and the age of two, which enabled us to characterize individual HC, height, and weight development with high precision and fit a negative exponential growth model to the data of each toddler with exceptional accuracy.

**Results:**

The analyses revealed that HC sizes and growth rates were not significantly larger in toddlers with autism even when stratifying the autism group based on verbal capabilities at the time of diagnosis. In addition, there were no significant correlations between ADOS scores at the time of diagnosis and HC at any time-point during the first 2 years of life.

**Conclusions:**

These negative results add to accumulating evidence, which suggest that brain volume is not necessarily larger in toddlers who develop autism. We believe that conflicting results reported in other studies are due to small sample sizes, use of misleading population norms, changes in the clinical definition of autism over time, and/or inclusion of individuals with syndromic autism. While abnormally large brains may be evident in some individuals with autism and more clearly visible in MRI scans, converging evidence from this and other studies suggests that enlarged HC is not a common etiology of the entire autism population. Early HC measures, therefore, offer very limited clinical utility for assessment of autism risk in the general population.

**Electronic supplementary material:**

The online version of this article (doi:10.1186/s13229-017-0129-9) contains supplementary material, which is available to authorized users.

## Background

Early brain overgrowth is one of the most prominent contemporary theories of autism development [1–3]. According to the theory, autism may be caused by different genetic predispositions and/or environmental insults that accelerate cellular proliferation, migration, differentiation, and development so as to generate abnormally large brains during the first 2 years of life. Early accelerated growth is thought to be followed by arrested growth, which explains why adolescents and adults with autism do not have larger brain volumes [4]. Nevertheless, the availability of head circumference (HC) as a biomarker during early development could be extremely useful for identifying toddlers at risk of developing autism even before the onset of behavioral symptoms [2]. Since HC is an excellent correlate of brain volume during the first years of life [5], testing whether toddlers with autism indeed exhibit early brain overgrowth is a relatively straight forward venture.

Previous studies have reported that toddlers who later develop autism are born with normal HC and then develop abnormally large HC during the first 2 years of life [6–10]. These findings have been corroborated by post mortem [11] and MRI [12–16] studies that have reported significantly larger brains in toddlers who develop autism. Later studies, however, have questioned whether early overgrowth is specific to the brain or reflects general body overgrowth as apparent also in height and weight measurements [17]. Furthermore, the validity of early findings that were mostly based on comparisons with CDC population norms have been questioned, because these norms have underestimated the true HC distribution in the general population [18]. With this in mind, several comparisons of early HC measurements between toddlers with autism and community-matched controls did not find any significant between-group differences [19–23]. These studies demonstrate the ongoing controversy regarding the existence and clinical significance of early brain overgrowth in toddlers with autism.

When interpreting the studies above, it is important to consider the sample characteristics in each case. For example, it has been suggested that larger brain volumes may be more strongly associated with specific autism etiologies involving regression [24], immunological insults [25], and specific genetic abnormalities (e.g., PTEN mutations [26]). Furthermore, HC is hereditary regardless of autism [27]. Differences in genetics and the environmental exposures of the examined sample as well as the inclusion/exclusion criteria of each study may, therefore, have an impact on potential differences across autism and control groups. For example, contrary to previous reports from the USA [28], assessment of large clinical databases in Norway and Israel reported modest [22] or no [29] differences in the rates of macrocephaly (HC >97% of the general population) in children diagnosed with autism.

To further address these issues, we performed a retrospective assessment of HC measurements that were recorded at birth and between the ages of 1–24 months at Maccabi Healthcare infant wellness centers in the southern district of Israel. The collected data included, on average, >8 HC, weight, and height measurements from each of the 66 toddlers who were later diagnosed with idiopathic autism and the 66 community-matched controls. This data contained approximately twice as many samples per toddler in comparison to previous studies. The large number of samples enabled us to examine the longitudinal HC development of individual toddlers with high temporal resolution and fit a negative exponential growth model to the data of each toddler with remarkable accuracy. In addition, we examined the potential relationship between early HC measures and autism severity measures at the time of diagnosis as assessed by the Autism Diagnostic Observation Schedule (ADOS; [30]). Importantly, the examined data represents an unbiased community sample of the members of Maccabi Healthcare services who make up approximately one third of the population in southern Israel.

## Methods

### Participants

We collected retrospective HC, weight, and height data from electronic patient records of children who were diagnosed with autism at the Maccabi Child Development Center in Beer Sheva (*n* = 66, 60 boys). The autism group included 22 children who were diagnosed according to DSM-IV criteria (17 with autism, 4 with PDD-NOS, and 1 with Asperger’s syndrome) and 44 children who were diagnosed according to DSM-V criteria and received a formal diagnosis of autism spectrum disorders (ASD). Diagnosis was confirmed in the first group of 22 children using the first [30] edition of the ADOS and in the later 44 children using the second [31] edition of the ADOS. The mean age of diagnosis was 36.94 months (standard deviation 13 months). Each child with autism was matched, in terms of gender and date of birth (±30 days), with a typically developing control child from the same southern Maccabi Healthcare district (Table 1). Children with known chromosomal disorders, known genetic disorders (e.g., RETT), hydrocephalus, additional developmental and neurological disorders, and those born before 36 weeks of gestation or below 2 kg were excluded from the study.

**Table 1:** Sample characteristics including the number of toddlers (relative number of females), the term in weeks, ADOS and ADI scores, age at diagnosis, and number of HC samples available from the individual patient records

	Autism	Control
Number of toddlers (females)	66 (6)	66 (6)
Term in weeks (range)	39.1 (36–42)	39.5 (36.2–41.5)
ADOS total (range)	16.1 (7–26)	
ADOS severity score (range)	7 (4–10)	
ADI-R (range)	18 (7–26)	
Age at diagnosis (range)	3 (1–5.3) years	
Number of HC samples	8.3 (4–12)	8.4 (4–12)

Range of the sample is in parentheses

### Head circumference, weight, and height data

We extracted all of the available HC, weight, and height measurements from the electronic patient records of each child. All of the measurements were performed by nurses at Maccabi Healthcare Infant Wellness Centers, except for the measurement at birth, which was conducted by a midwife. Both the nurses and the midwives perform dozens of HC, weight, and height measurements every week and are extremely proficient in this work. The mean number of measurements per toddler was 8.3 (SD 2.1) in the ASD group and 8.42 (SD 1.74) in the control group (range 4–12 samples per child in both groups). There were no significant differences in the number of measurements across ASD and control groups (*t* (125) = −0.48, *p* = 0.6, two-tailed *t* test). In Israel, all children are invited to visit a baby clinic at the ages of 1, 2, 4, 6, 9, 12, 15, 18, and 24 months to complete standard check-ups and immunizations as part of the free social health care services. The final number of visits/measurements and the precise age of the visits, however, depend on parental compliance.

### Data analyses

**Fig. 1:**
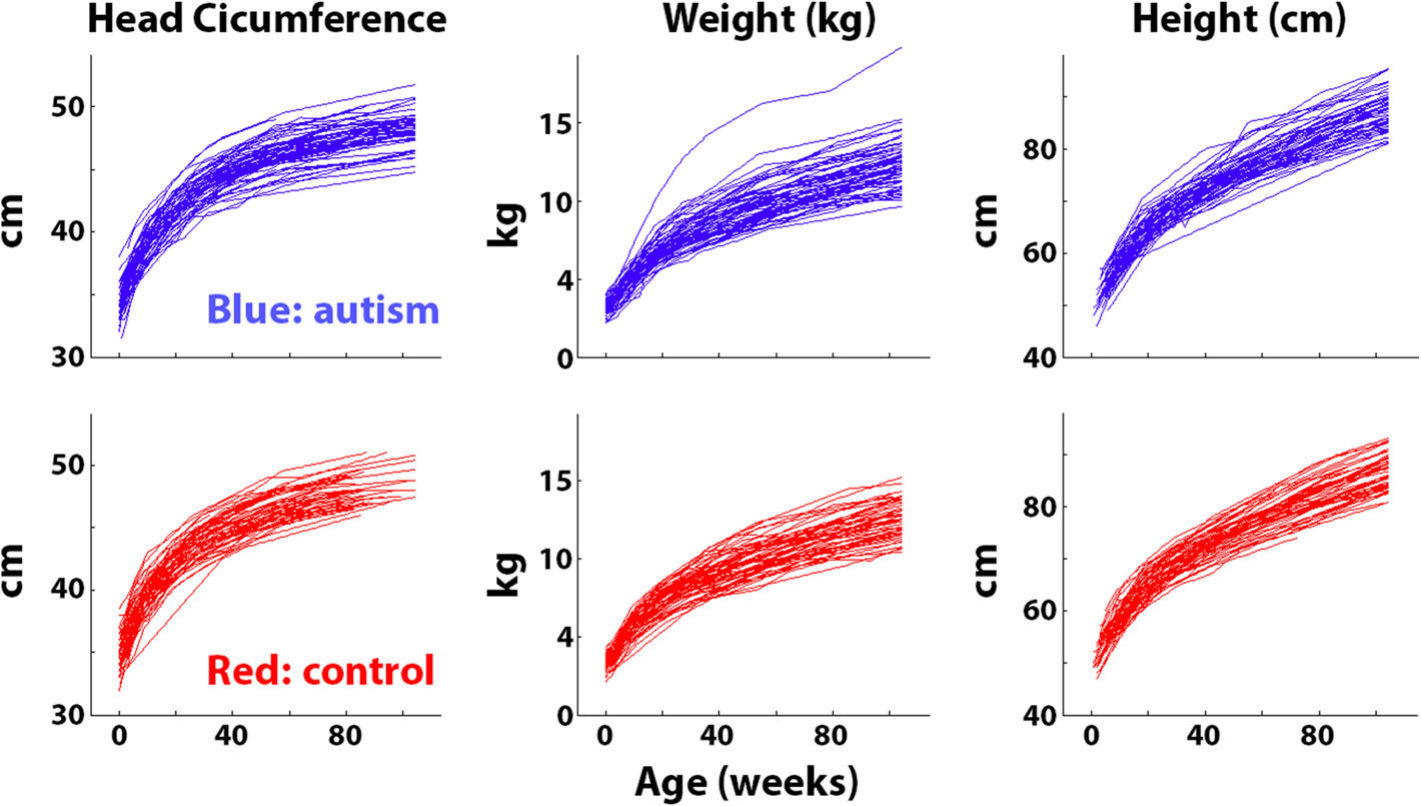
Head circumference, weight, and height measurements from the 66 toddlers who later developed autism (*blue*) and the 66 matched controls (*red*). Each *line* represents the development of a single toddler as estimated with linear interpolation between measurement points (see “Methods”)

Analyses were performed with custom written code in Matlab (Mathworks Inc., USA). Since each child had measurements at different time-points within the first 24 months of life, we first linearly interpolated the measurements into vectors with a time resolution of weeks using the *interp1* function in Matlab. This enabled us to estimate the week-by-week HC, weight, and height values of each child from the age of their first measurement to the age of their last measurement without assuming an overall growth model with a predefined shape (Fig. 1). We then compared HC, weight, and height of children who later developed autism and controls while binning the data into 3-month intervals from birth to the age of 24 months. We used two-tailed two-sample *t* tests with unequal variances to assess the significance of differences across groups. We also computed the Pearson’s correlation coefficient between ADOS severity scores (see below) and HC measurements for each of the age intervals (bins) described above. The significance of the correlation coefficients was not corrected for multiple comparisons in order to increase sensitivity.

ADOS calibrated severity scores (also known as “Comparison Score”) were calculated using the ADOS-2 algorithm [31] in children who were diagnosed by the ADOS-2 (*n* = 44) or an alternative algorithm [32] available for children who were diagnosed with the first version of ADOS (*n* = 22).

We used a negative exponential growth model (Matlab code is available in the Additional file 1) that was used in previous HC studies of autism [19] to estimate the rate of HC growth in each toddler during the first 2 years of life:

$$ \mathrm{H}\mathrm{C} = \alpha +\left(\beta -\alpha \right)*{e}^{-\gamma \ast x} $$

The model describes a nonlinear function where *α* represents the asymptote (a maximum size for growth within the time frame considered), *β* represents the intercept at age 0 (i.e., birth), and *γ* represents the anti-log of the rate of change (growth rate from birth to the asymptote). The three parameters were estimated for each of the toddlers. We used the same model to examine weight and height growth as well. We tested for group differences by performing two-tailed *t* tests with unequal variances for each of the model parameters. The significance of differences across groups was not corrected for multiple comparisons in order to increase sensitivity.

## Results

### Individual development curves

On average, there were >8 HC, height, and weight measurements available for each toddler in our sample. The precise number of measurements and their relative timing (i.e., toddler’s age at each measurement), however, varied across toddlers. To overcome these differences across toddlers, we performed a linear interpolation over time, connecting pairs of datapoints with straight lines. In this manner, we built a growth curve for each toddler with a temporal resolution of weeks (Fig. 1). This enabled us to examine the developmental changes in HC, weight, and height of individual toddlers from each of the groups throughout their first 2 years of life. All three measures exhibited the typical logarithmic shape, and the distribution of individual values was qualitatively similar across the two groups (Fig. 1). Note the high-resolution of these linearly interpolated growth curves, which is a feature of the large number of samples obtained from each toddler (see “Methods”).

### Comparisons across autism and control groups

**Fig. 2:**
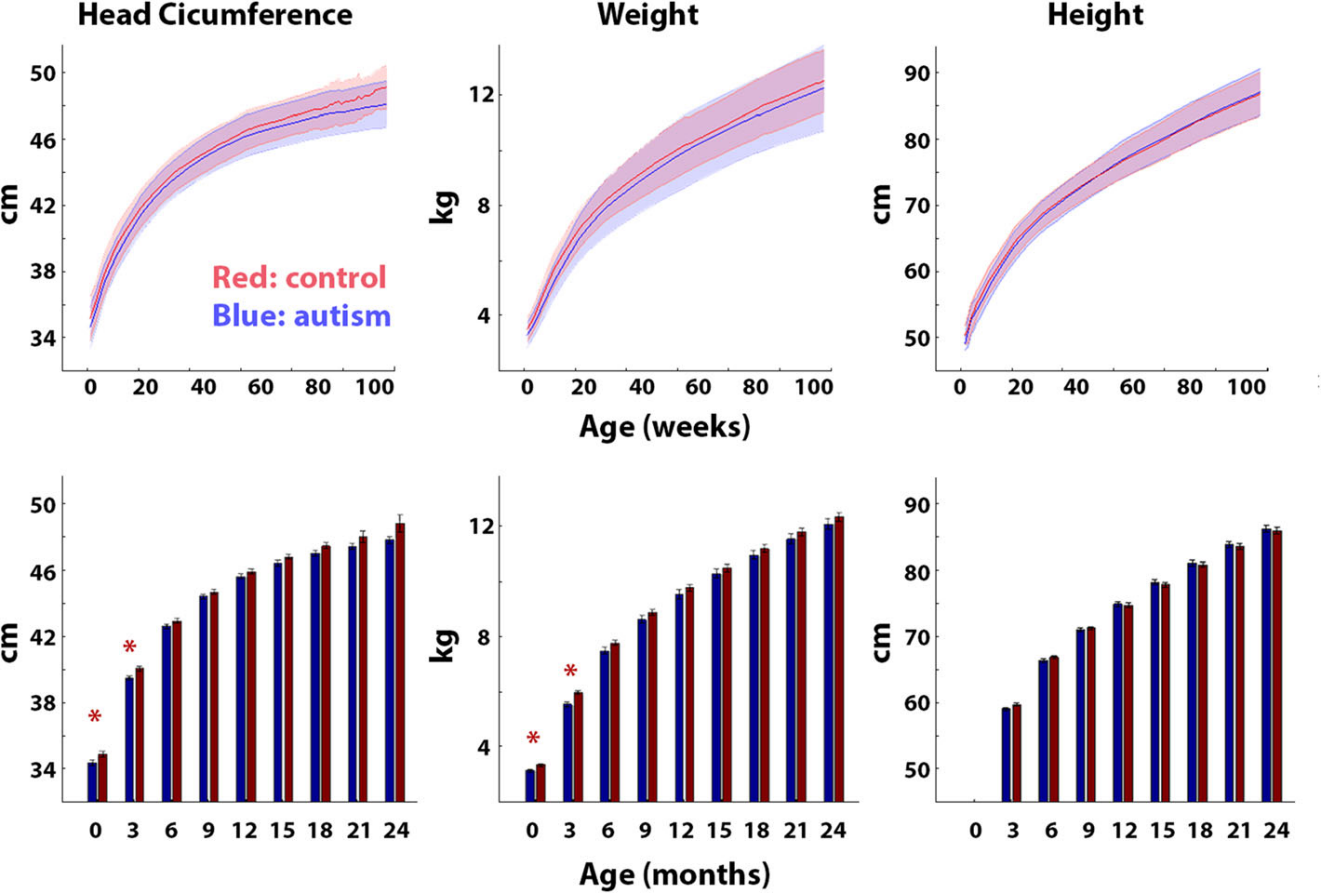
Comparison of HC, weight, and height across autism (*blue*) and control (*red*) groups. *Top row*: Mean of each group as a function of age in weeks. *Shaded area*: standard deviation across toddlers. *Bottom row*: Mean of each group in 3-month periods. *Error bars*: standard error of the mean across subjects. *Red asterisks*: significantly larger values in the control group (*p* < 0.05, uncorrected to increase sensitivity)

Mean growth curves of the two groups were overlapping for all three measures (Fig. 2, top row). We tested whether there were any differences across groups at multiple ages between birth and 24 months in 3-month intervals. Note that height is not measured at birth in Israel; hence, comparisons of this measure start from the age of 3 months. The control group exhibited significantly larger head circumference and weight at birth and at 3 months of age (*p* < 0.05, *t* > 2.1, uncorrected to increase sensitivity, Fig. 2, bottom row). All other between-group comparisons did not reveal any statistically significant differences across groups (see Table 2). Note that with 66 toddlers in each group, our sample size yields a power of 0.88 for detecting larger HC measures in the autism group (assuming an effect size of 0.5).

**Table 2:** Comparisons of HC, weight, and height across groups from birth to the age of 24 months in 3-month intervals

Head circumference					
Age	Autism	Control	Autism-control	*t* value	*p* value
Birth	34.4	35	−0.6	−2.1	0.04*
3 months	39.7	40.2	−0.5	−2.8	0.006*
6 months	42.8	43.2	−0.4	−1.7	0.1
9 months	44.7	44.9	−0.2	−1.3	0.19
12 months	45.9	46.2	−0.3	−1.3	0.21
15 months	46.7	47.1	−0.4	−1.7	0.1
18 months	47.2	47.7	−0.5	−1.9	0.06
21 months	47.7	48.3	−0.6	−1.4	0.17
24 months	48.1	49.1	−1	−1.8	0.12
Weight					
Age	Autism	Control	Autism-control	*t* value	*p* value
Birth	3.2	3.4	−0.2	−2.7	0.007*
3 months	5.6	6.1	−0.5	−3.8	0.0004*
6 months	7.6	7.9	−0.3	−1.9	0.06
9 months	8.7	9	−0.3	−1.4	0.1
12 months	9.7	9.9	−0.2	−1.2	0.24
15 months	10.4	10.6	−0.2	−0.9	0.36
18 months	11.1	11.3	−0.2	−1	0.31
21 months	11.7	12	−0.3	−1.1	0.28
24 months	12.3	12.5	−0.2	−1	0.31
Height					
Age	Autism	Control	Autism-control	*t* value	*p* value
3 months	59.4	60	−0.6	−1.9	0.06
6 months	66.9	67.3	−0.4	−1.2	0.24
9 months	71.5	71.8	−0.3	−1.4	0.58
12 months	75.5	75.3	0.2	−1.2	0.73
15 months	78.8	78.5	0.3	−0.9	0.53
18 months	81.7	81.5	0.2	−1	0.72
21 months	84.5	84.2	0.3	−1.1	0.63
24 months	87	86.7	0.3	−1	0.67

HC and height are in centimeters and weight is in kilograms. Columns: mean of the autism group, mean of the control group, the difference between autism and control groups, *t* values, and *p* values from a two-tailed two-sample *t* test with unequal variance. *p* values are not corrected to increase sensitivity

Asterisks: *p* value < 0.05

### Comparison of growth model parameters

**Fig. 3:**
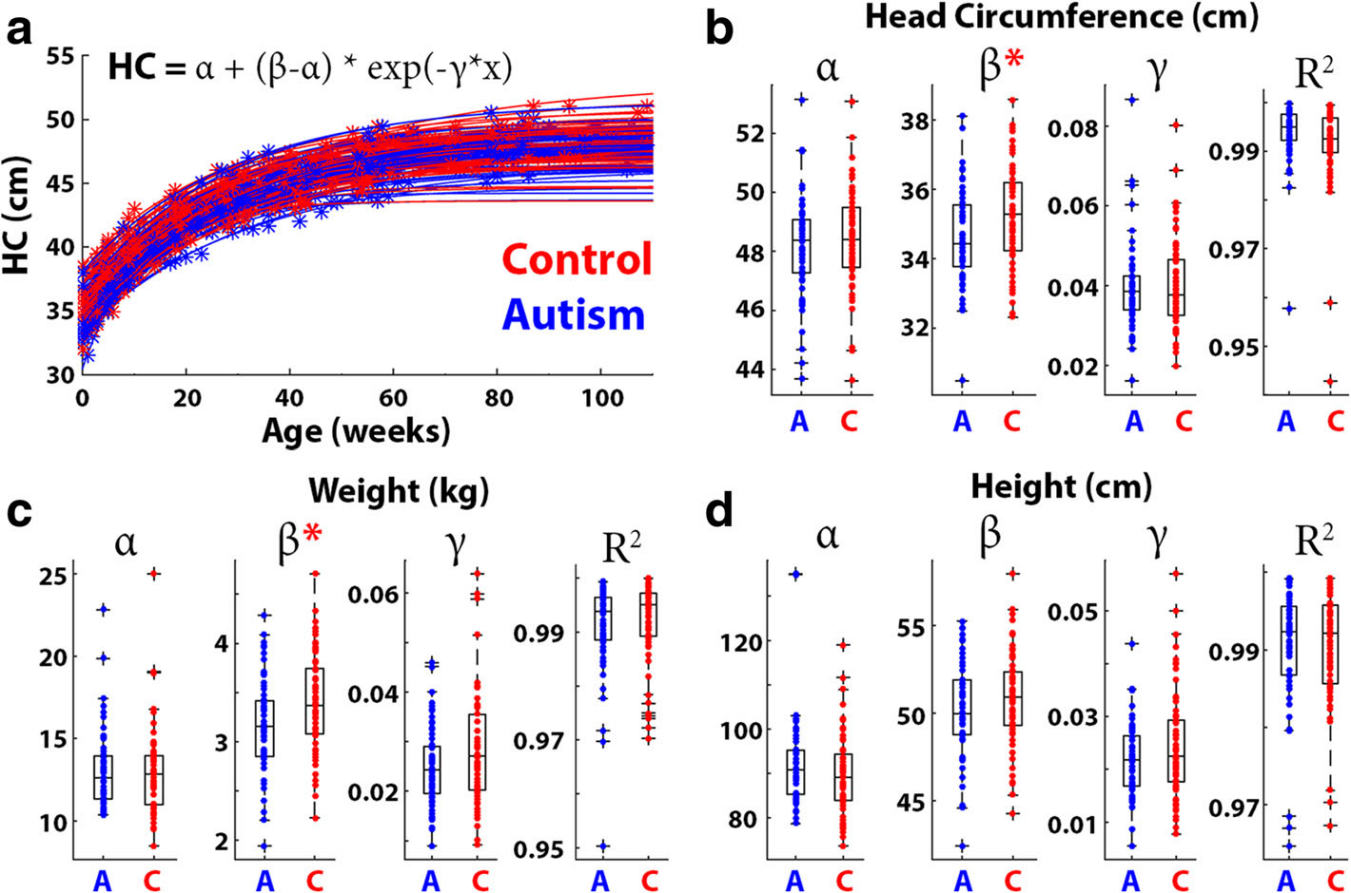
Comparison of head growth modeling parameters across autism (*blue*) and control (*red*) groups. Demonstration of HC growth model fits in individual toddlers: *asterisks* represent measurements and the *lines* represent the model fits (**a**). *Boxplots* demonstrating the HC (**b**), weight (**c**), and height (**d**) growth parameters: asymptote (*α*), intercept (*β*), and rate of change (*γ*) in each group. *Red asterisks*: significantly larger parameter values in the control group. *R* ^2^: *box plots* demonstrating the model fits of individual toddlers in each group for each of the measures

We fit a nonlinear growth model (see “Methods”), which contained three parameters: intercept, rate of change, and asymptote, to the data of each toddler (Fig. 3). In agreement with the results described above, control toddlers exhibited significantly larger intercept (*β*) values when modeling HC (*p* = 0.02, *t* = 2.3, Fig. 3b) or weight (*p* = 0.007, *t* = 2.8, Fig. 3c) measures. The significant difference in the intercept indicates that HC and weight measures were significantly larger in control toddlers at birth. The two other parameters did not differ across groups for any of the measures. The lack of difference in rate of change indicates that there were no significant growth rate differences between autism and control toddlers in any of the measures. Note that the model fit the data of individual toddlers extremely well, explaining >94% of the variance in all cases, for all three measures (*R* ^2^ values in Fig. 3).

Performing the same analysis only with male toddlers produced equivalent results: control toddlers exhibited significantly larger intercept (*β*) values when modeling HC (*p* = 0.03, *t* = 2.2) or weight measures (*p* = 0.01, *t* = 2.5), and there were no significant differences across groups in the rate of change and asymptote parameters.

### Head circumference and autism severity

**Fig. 4:**
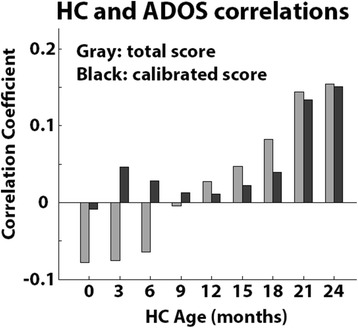
Relationship between HC at specific ages and ADOS score at time of diagnosis. Pearson’s correlation coefficients were computed for HC and total ADOS (*gray*) scores or calibrated ADOS scores (*black*)

We examined the potential relationships between HC as measured at specific ages and autism severity as quantified by the ADOS total score or calibrated severity score (see “Methods”) at the time of diagnosis. All of the correlations were weak and not statistically significant indicating that HC measures were not associated with ADOS scores (Fig. 4).

### Verbal and nonverbal toddlers

**Fig. 5:**
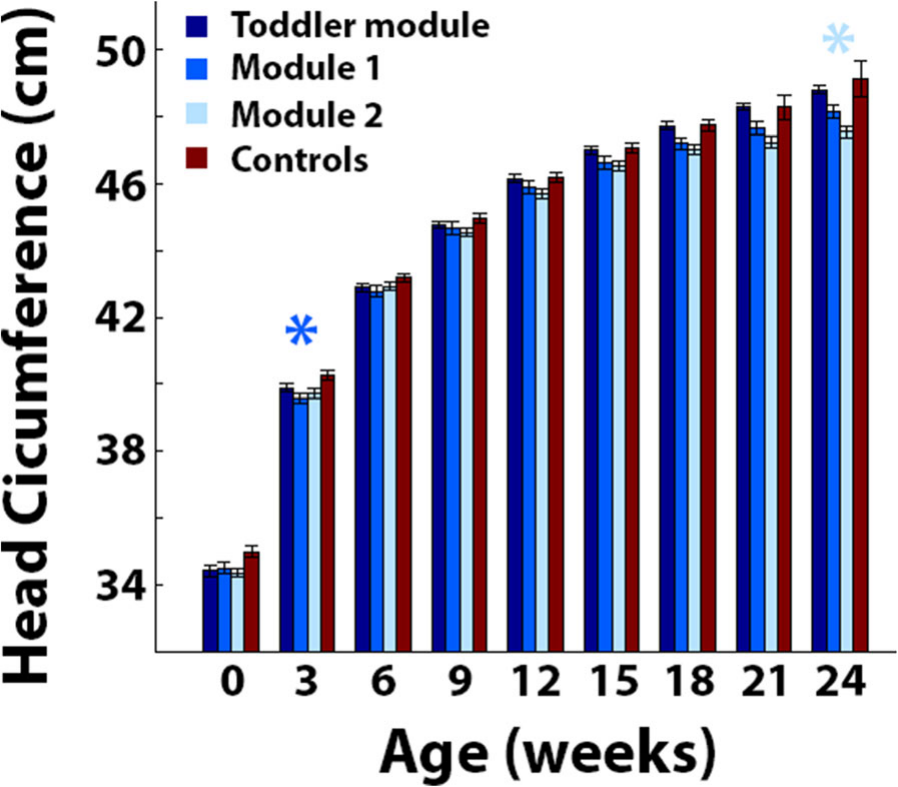
Comparison of HC at specific ages when separating the autism group according to ADOS modules. Mean HC in toddlers with autism diagnosed with the toddler module (*dark blue*), module 1 (*medium blue*), or module 2 (*light blue*), and control toddlers (*red*). *Error bars*: standard error of the mean across toddlers in each group. *Asterisks*: significantly larger HC in the control group as compared with that in the autism group of the corresponding color (*p* < 0.05, uncorrected to increase sensitivity)

We performed the same HC comparisons described above (Fig. 2), while separating nonverbal toddlers who were diagnosed with the toddler module (*n* = 14) or module 1 (*n* = 36) of the ADOS from the verbal toddlers who were diagnosed with module 2 (*n* = 15) of the ADOS. Significantly larger HC was apparent in the control group at 3 months of age in comparison to the autism toddlers diagnosed with module 1 (*p* = 0.008, *t* = 2.7, two-tailed *t* test, uncorrected to increase sensitivity) and at 24 months of age in comparison to the autism toddlers diagnosed with module 2 (*p* < 0.04, *t* > 2.4, two-tailed *t* test, uncorrected to increase sensitivity). All other differences between autism and control groups were not significant (Fig. 5). This indicates that early HC measures are not larger in children who later develop autism regardless of their verbal abilities at time of diagnosis.

## Discussion

Our results demonstrate that toddlers diagnosed with autism in southern Israel do not exhibit early HC overgrowth during the first 2 years of life. Comparisons with control toddlers who were born at equivalent times and geographical locations revealed that HC, weight, and height measurements were mostly indistinguishable from those of toddlers with autism during the first 24 months of development and, if anything, were larger in the control group at birth and 3 months of age (Figs. 1 and 2). Furthermore, growth parameters estimated using a nonlinear negative exponential model were not significantly different across groups except for the intercept parameter, which indicated significantly larger HC and weight measures in the control group at birth (Fig. 3). Equivalent findings were evident when examining only male toddlers and when splitting toddlers with autism into verbal and nonverbal groups according to the ADOS modules used for diagnosis (Fig. 5). Finally, HC during the first 24 months of development did not predict later autism severity at the time of diagnosis (Fig. 4).

An important strength of the current study lies in the relatively large number of measurements that were extracted from each of our 132 participants. While most previous studies relied on 3–4 HC samples per child, we extracted >8 HC, weight, and height measurements per toddler, on average. This enabled us to estimate HC, weight, and height growth rates with exceptional accuracy as reflected in the goodness-of-fits of individual toddlers (>94% of the variance explained in all toddlers, Fig. 3). Furthermore, each of the 66 toddlers with autism was closely matched to a control toddler who was born within 30 days in the same geographical location. This ensured that the examined HC, weight, and height measurements were collected by similarly trained clinical staff within the same local community. Hence, the growth rates presented in the current study are likely to represent the true distribution of rates in the autism and control populations of southern Israel.

### HC overgrowth in autism

Previous studies have reported that HC is enlarged in autism during the first 2 years of life [6–10, 12, 17]. Analogous studies have reported significantly higher rates of macrocephaly (HC >97% of the general population) in children with autism [33–35]. While many of the HC and macrocephaly studies described above were biased by the use of misleading population norms published by the CDC [18], some studies have reported significantly larger HC in autism, even in comparisons with control toddlers from the same community [12, 17]. This evidence is often cited in support of the prominent early overgrowth theory of autism, which suggests that autism may be caused by abnormal cellular proliferation, migration, and differentiation that generate accelerated brain growth during the first 2 years of life [1–3].

In contrast to the studies described above, a growing body of literature has reported that HC measures during the first 2 years of life are not significantly larger in toddlers who later develop autism [19–23]. Similarly, recent assessments of large clinical databases in Norway and Israel have reported modest [22] or no [29] difference in macrocephaly rates between autism and control populations. Our results are in line with these later studies and present further evidence for a lack of difference across groups in both HC size and growth rate during the first 2 years of life.

We believe that the conflicting results between older studies that reported significant HC differences across groups and more recent studies that do not may be explained by several key reasons: First, many of the earlier studies compared HC measurements from toddlers with autism to outdated and misleading CDC norms that underestimated the true HC distribution of the general population in the USA [18]. Second, earlier studies, even if they included matched controls, were based on small samples of 20–30 individuals in each group while more recent studies and the current one were based on sample sizes that were at least twice as large. Given the large variability in HC across individuals of each group (Figs. 1 and 2), studies with larger samples are more likely to yield accurate estimates of the HC distributions in each group, which are necessary for assessing differences across groups [4]. Third, older studies may have included subjects with known genetic syndromes in comparison to more recent studies that exclude such individuals. Since syndromal subtypes of autism are specifically associated with macrocephaly (e.g., tuberous sclerosis [36] and PTEN mutations [26]), it is possible that some of the older studies may have included rare participants with extreme HC measures.

Another potential reason for conflicting findings across older and newer studies is the ongoing change in clinical criteria of autism diagnoses, which have led to a considerable increase in autism prevalence [37]. For example, recent samples of children with autism are likely to include children with milder forms of autism and/or higher cognitive function [38] as well as children who would have received a mental retardation diagnosis in the past [39]. While we did not find a relationship between early HC measures and autism severity at the age of diagnosis (Fig. 4), an older study did report such a relationship [9]. In addition, given the dramatic increase in autism prevalence, current samples of children with autism are likely to contain more biological heterogeneity and relatively fewer syndromic cases of autism.

### Environmental and genetic factors associated with HC growth

HC growth rates are dependent on a wide variety of environmental and genetic factors, regardless of autism. For example, HC growth is associated with the richness of postnatal nutrition [40], pre-natal exposure to a variety of substances including folic acid [41] and acrylamide [42], and maternal stress levels during pregnancy [43]. Genetic risk factors include polymorphisms and mutations in a variety of genes such as PTEN and CHD8 [44]. Furthermore, HC is highly heritable regardless of autism and is highly correlated with genetic ancestry [27].

This means that the utility of early HC measures for clinical assessment of autism risk depends on whether findings are generalizable across multiple populations with distinct genetics and environmental exposures. The negative findings in this study and those of a recent macrocephaly study in the Israeli population [29] suggest that HC is, at the very least, a poor predictor of autism risk in the Israeli population. The equivalent negative results reported in studies from North America [19–21] and Europe [22], however, suggest that our findings are not due to genetic and/or environmental factors that are unique to Israel. A clearer understanding of how brain overgrowth differs in samples with distinct genetic and environmental factors is, therefore, critical for further evaluating the clinical utility of early HC measures in specific sub-groups of children with autism.

### The overgrowth hypothesis of autism

HC is obviously a very coarse measure for testing the overgrowth hypothesis of autism. MRI scans offer far more detailed and accurate measures of total brain volume while enabling separation of gray and white matter volumes and assessment of cortical thickness and surface area. While such measures do not differ significantly between autism and control subjects who are over the age of six [4], toddlers with autism may exhibit larger brain volumes during the second year of life in comparison to controls [12–16]. Furthermore, specific overgrowth of cortical surface area may be apparent even during the first year of life in those who later develop autism [16]. Additional evidence from diffusion tensor imaging (DTI) studies suggests that toddlers with autism exhibit abnormal white matter microstructure, which may indicate early over-proliferation of neurons [45, 46] as also found in several post mortem studies [11, 47].

While specific neuroimaging measures of brain overgrowth may indeed differ between individuals with autism and controls during early development, converging evidence seems to suggest that HC measures do not capture these differences in a reliable manner during the first 2 years of life. This is unfortunate because neuroimaging studies are expensive and examine small samples that are usually biased (i.e., usually include parents who have the time, motivation, and capabilities to bring their children to the MRI center). In contrast, HC studies are cheaper and enable assessment of larger unbiased samples from the general population by examining HMO databases.

## Conclusions

Given the large heterogeneity of mechanisms that have been implicated in the development of autism [48], it is not surprising that there are mixed reports regarding the existence of HC differences across autism and control groups in different studies/samples. Indeed, similarly conflicting reports exist with respect to many physiological and behavioral measures studied in individuals with autism [49]. The great challenge facing contemporary autism research is to define specific sub-groups of toddlers with autism who exhibit specific etiologies. While early brain overgrowth may embody one such etiology, it is likely to be relevant to particular syndromic forms of autism and to specific individuals who exhibit abnormally large brain volumes early in life. Future research regarding early overgrowth would, therefore, benefit from targeted studies with these particular individuals rather than attempting to associate early overgrowth with the entire autism population.

## Additional file

Additional file 1: Matlab code for fitting the negative exponential growth model. (DOCX 15 kb)
