# Supplementary material for: No evidence of early head circumference enlargements in children later diagnosed with autism in Israel
Source: Mol Autism. 2017 Mar 23;8:15. doi: 10.1186/s13229-017-0129-9 (PMC5363048; doi:10.1186/s13229-017-0129-9)
Supplement: Additional file 1: — Matlab code for fitting the negative exponential growth model. (DOCX 15 kb) [file 13229_2017_129_MOESM1_ESM.docx]

**No evidence of early head circumference enlargements in children later diagnosed with autism in Israel – Supplementary Materials**

Ilan Dinstein^1,2,*^, Shlomi Haar^2^, Shir Atsmon^2^, Hen Schtaerman^3^

1. Psychology Department, Ben Gurion University, Beer Sheva, Israel, 84105
2. Cognitive and Brain Sciences Department, Ben Gurion University, Beer Sheva, Israel, 84105
3. Child Development Center, Maccabi Health Services, Beer Sheva, Israel, 84893

* Corresponding author: Ilan Dinstein, [dinshi@bgu.ac.il](mailto:dinshi@bgu.ac.il), +972864672071

**Matlab code for fitting the negative exponential growth model:**

The code below demonstrates the HC analysis in 66 toddlers with autism. The code
requires a cell array named ASD_data, which has one cell for each child which include a matrix with two columns: The first column contains the HC measurements of the child, and the second contains the age in weeks of each measurement.

Our growth model contained three parameters – A: asymptote (final HC at the age of 24 months), B: intercept (HC at birth), and C: negative exponential growth rate (change in HC over time).

We used the matlab function fmincon to find a solution for each of the three parameters described above that minimized the root mean square (rms) error between the model and the data (using the Optimization function shown below).

global data

num_subjects = length(ASD_data);

for sub=1:num_subjects

data = ASD_data{sub}; % extract the toddler’s data into the global variable 'data'

used by the Optimization function below

[p, fval]=fmincon(@Optimization,[50 35 0.05],[0 0 0],1);

% The fmincon function performs an iterative search for a minimum value given % a predefined arbitrary starting point for each of the three parameters, which % we set to [A B C] = [50 35 0.05]. The selected starting point did not matter as
% we reached the same solutions when applying any three values here.

% p = the values of the three parameters that minimize the rms error.
% fval = the minimal value of the rms error.

% We then computed the fit of the model to the data as follows:

R2 = corr(p(1)+(p(2)-p(1))*exp(-p(3)*data(:,2)),data(:,1))^2;

HC_ASD(sub,:)=[p R2];

end

function rms = Optimization(param)

global data

A = param(1); B = param(2); C = param(3);

HC = A+(B-A)*exp(-C*data(:,2));

rms = sqrt(mean(((HC-data(:,1)).^2)))/sqrt(numel(HC));
